# Soil C, N, P and K stoichiometry affected by vegetation restoration patterns in the alpine region of the Loess Plateau, Northwest China

**DOI:** 10.1371/journal.pone.0241859

**Published:** 2020-11-05

**Authors:** Ruosha Liu, Dongmei Wang

**Affiliations:** College of Soil and Water Conservation, Beijing Forestry University, Beijing, China; Emory University, UNITED STATES

## Abstract

The Grain-for-Green project is an important ecological restoration measure to address the degradation of alpine ecosystems in China, which has an important impact on the ecological stoichiometry of soil carbon (C), nitrogen (N), phosphorus (P) and potassium (K). However, soil stoichiometry changes under different vegetation restoration patterns and at different soil depths remain poorly understood in the alpine region of the Loess Plateau. To clarify these soil stoichiometry changes, a 0–60 cm soil profile was sampled from two typical vegetation restoration patterns: grassland (GL) and forestland (FL), including *Picea crassifolia* (PC), *Larix principis-rupprechtii* (LR), *Populus cathayana* (PR) and *Betula platyphylla* (BP). The control was a wheat field (WF). In all soil layers, the soil organic carbon (SOC), total nitrogen (TN), soil available nitrogen and potassium (AN and AK, respectively) and C:P, C:K, N:P and N:K ratios of FL were higher than those of GL and WF. The TN content and N:P and N:K ratios of GL were higher than those of WF in each soil layer. Additionally, the soil nutrients (except TK) of all vegetation types and stoichiometry of PR and GL (except the N:P ratio of GL) were greater at 0–20 cm than at 20–60 cm. Moreover, the SOC and TN showed the strongest correlation with the soil stoichiometry (except P:K ratio); thus, C and N had the greatest effect on the soil stoichiometry. Furthermore, soil fertility was limited by N. Our results indicated that different vegetation restoration patterns and soil depths had significant effects on the soil nutrients and stoichiometry in the alpine region of the Loess Plateau. The recovery of farmland to forestland promoted better improvements of soil nutrients, and PR had the most significant positive effect on soil surface nutrients.

## Introduction

As the largest carbon (C) pool in the terrestrial biosphere, soil plays an important role in the global C cycle [1]. Nitrogen (N) and phosphorus (P) are important elements for organisms [2, 3], and potassium (K) is related to the metabolism of organisms [4]. Soil N, P and K in terrestrial ecosystems are closely related to terrestrial biogeochemical cycles and have significant effects on the primary yield and C accumulation [5]. The contents and ratios of C, N, P and K in soil can directly affect the absorption and utilization of these elements by plants and even change the overall biomass allocation and ecological strategies of plants [6]. Therefore, the study of soil C:N:P:K stoichiometry, particularly the N:P ratio, contributes to understanding the biogeochemical processes, nutrient cycling and nutrient limits in terrestrial ecosystems [7].

Currently, many studies have been carried out on soil ecological stoichiometry at a global scale to determine the soil ecological stoichiometry changes under different latitudes, depths and land uses as well as the interaction with soil microorganisms [8–12]. For example, Zhang [13] concluded that the content of C and N and the ratio of C:P and N:P in the soil were lower at high altitudes. Brady and Weil [14] found that the total P content presented a complex and highly variable vertical pattern throughout the soil profile. Zhang [12] indicated that the soil C:P and N:P ratios decreased with increasing latitude, with spatial patterns being primarily regulated by climate factors. These studies have deepened our understanding of the geographical and spatial patterns and influencing factors of soil ecological stoichiometry. With the strengthening of global environmental protection, soil ecological stoichiometry has become one of the research hotspots in restoration ecology [15]. The research contents mainly focus on the differences of soil nutrients (or reserves) and ecological stoichiometry for different restoration processes, restoration years and land use types [16–18], whereas few studies have focused on the soil stoichiometry of different vegetation restoration patterns.

Vegetation restoration refers to the conversion of nonvegetated or cultivated land to vegetation cover, which is an effective measure to repair a damaged natural ecosystem [19, 20]. Studies have confirmed that vegetation restoration has a significant impact on soil physical and chemical properties, vegetation characteristics, C and N cycles, and land management [21, 22]. For instance, Zhao [23] and Fu [24] showed that the contents of soil organic C (SOC), soil total N (TN) and soil total P (TP) increased significantly after cropland was restored to artificial forestland and grassland. Jiao [25] and Li [26] found that replanting eroded soil could increase the SOC, TN and TP contents in soil. To control soil erosion, restore vegetation and improve the environment in China, the Chinese government initiated the Grain-for-Green project in 1999 and carried out large-scale vegetation restoration and reconstruction on steep slopes (>25°) using trees, shrubs or herbs [27, 28]. Studies have shown that the soil moisture, surface runoff, soil erosion and species diversity change after returning cropland to forestland [29]. For example, Zhang [30] found that the species richness and diversity increased after vegetation restoration. Mulder and Elser [31] showed that the soil acidity decreased after vegetation restoration. With the changes in species composition, biomass and soil characteristics that occur during the process of returning cropland to forestland, the cycles of C, N, P and K in soil may change significantly, thus affecting the succession and ecological processes of plants [32–34]. Therefore, studying the changes in soil nutrients and stoichiometry of different vegetation restoration models can clarify the restoration effect of different models and the evolution trend of soil fertility and provide a scientific basis for ecological restoration measures.

The alpine region of the Loess Plateau belongs to an alpine ecosystem [35]. Due to its high altitude and unique climatic conditions, the region suffers from poor soil nutrients, severe desertification and soil erosion; thus, it is a highly vulnerable ecological environment [36, 37]. To improve the ecological environment, the "Grain-for-Green Program" was implemented in 1999 to restore the local vegetation [38–40]. With the increase in global warming and N deposition, the storage of nutrients in the alpine ecosystem is becoming increasingly important. The soil in an alpine region can not only positively affect global carbon accumulation but also affect the functions of the regional ecosystem [41]. Therefore, studying the effects of different vegetation restoration patterns on soil C, N, P and K stoichiometry in alpine regions is of great significance to regional vegetation restoration and forest management. However, the changes in soil ecological stoichiometry and the distribution along the soil profile during the vegetation restoration process in alpine ecosystems are still unclear. Thus, the objectives of the present study were to (1) explore the characteristics of the variations in soil C, N, P and K contents and stoichiometry under different vegetation restoration patterns in the alpine region of the Loess Plateau; (2) clarify the profile distributions of soil C, N, P and K contents and stoichiometry; and (3) reveal the effects of different vegetation restoration patterns on soil nutrient restoration and the limiting elements of soil in the area.

## Materials and methods

### Ethics statement

The research site was not privately owned or protected in any way, and the field studies did not involve endangered or protected species. The field survey of the research site was allowed by the forestry station of Datong county, Qinghai Province, China.

### Study area

The study was conducted in Datong County, Xining City, Qinghai Province (36°43′-37°23′N, 100°51′-101°56′E, 2280–4622 m a.s.l.), which is located in the transitional zone between the western part of the Loess Plateau and the Qilian Mountains. The study area has a semiarid and semihumid plateau continental climate. There are 2605 annual sunshine hours. The frost-free period is 70–120 d throughout the year. The average annual temperature is 2.8°C, and the highest and lowest temperatures are 30.9°C and -33.1°C, respectively. The average annual precipitation is 508 mm, with 75% of the annual rainfall occurring in July, August and September. The soil is mainly mountain brown soil developed on loess parent material [42].

The territory is surrounded by mountains on three sides, the gullies are vertical and horizontal, the terrain is high in the northwest and low in the southeast, and soil erosion is severe. Since 1999, to control the local soil erosion problem, the Grain-for-Green project has been carried out to restore local vegetation [43]. The local shrub and herb species mainly include *Hippophae rhamnoides*, *Caragana korshinskii*, *Lycium chinense*, *Spiraea alpina*, *Elymus nutans* and *Equisetum arvense*. The main species planted in the study area are *Picea crassifolia*, *Sabina przewalskii*, *Larix principis-rupprechtii*, *Populus cathayana* and *Betula platyphylla*. The main agricultural land type in the research area is sloping land, the main crop is wheat, and the growth and development of crops mainly depend on natural rainfall and artificial fertilization (mainly inorganic fertilizer).

### Experimental design

**Fig 1:**
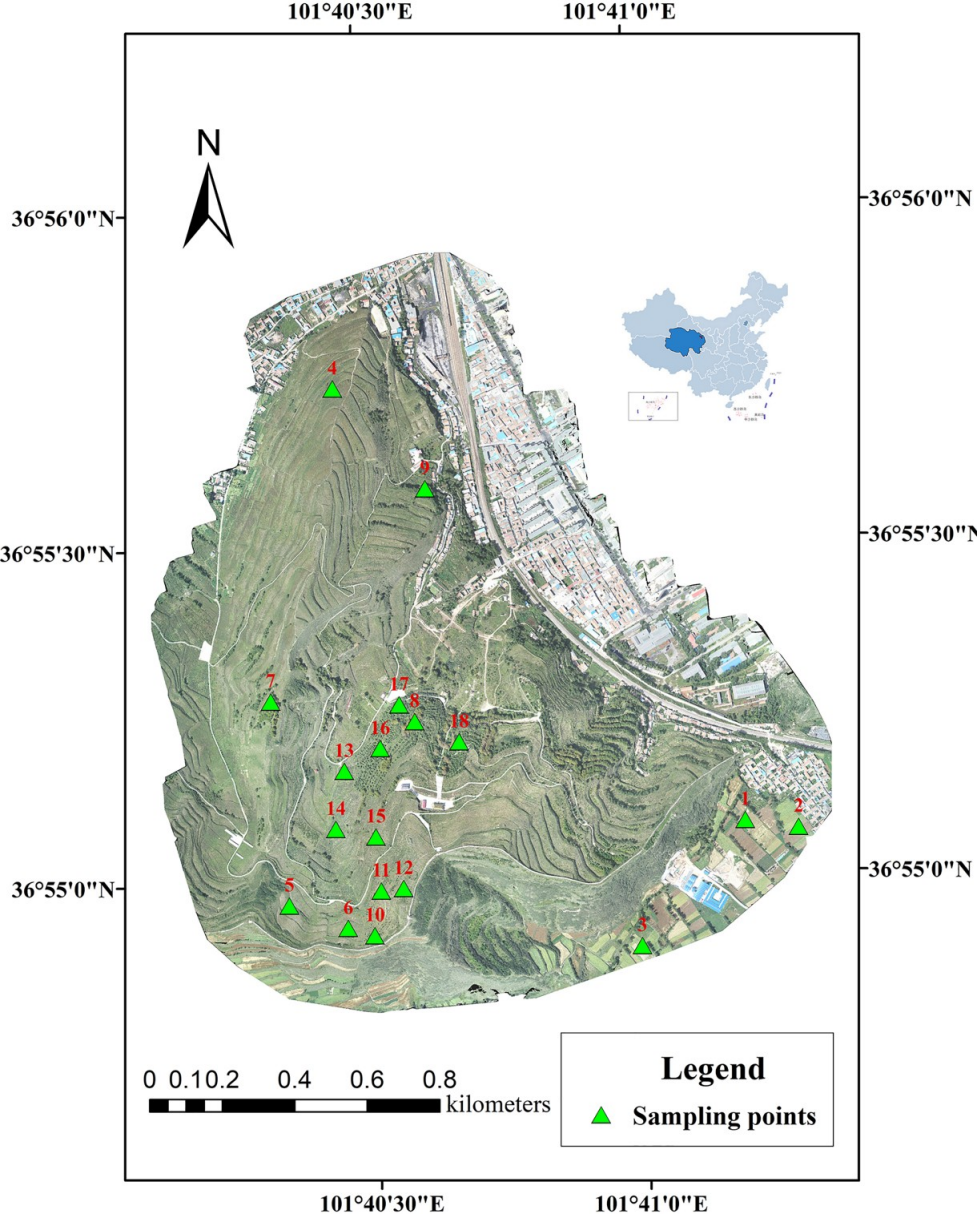
Location of the sampling sites in the alpine region of Loess Plateau. 1, 2 and 3 represent wheat fields; 4, 5 and 6 represent grasslands; 7, 8 and 9 represent *Populus cathayana* (PR); 10, 11 and 12 represent *Betula platyphylla* (BP); 13, 14 and 15 represent *Picea crassifolia* (PC); and 16, 17 and 18 represent *Larix principis-rupprechtii* (LR).

In this research, the artificial forestland (FL) and natural grassland (GL) that were converted from farmland at the Anmentan catchment were selected as the research objects. FL included *Larix principis-rupprechtii* (LR), *Betula platyphylla* (BP), *Picea crassifolia* (PC) and *Populus cathayana* (PR) for 20 years, and GL was abandoned cropland for 20 years. Wheat fields (WFs) near the site were selected for comparison. In June 2018, three sample plots were selected for each vegetation type in the research area, and three 30×30 m^2^ quadrats were selected for each sample plot for subsequent observation and sampling (Fig 1). The selected plots were similar in terrain (e.g., slope, aspect) and consistent in climate and other conditions. The soil chemical properties and nutrient content before vegetation restoration are shown in Table 1, and the basic information on each vegetation type is shown in Table 2.

**Table 1:** Soil chemical properties and nutrient content before vegetation restoration.

Soil layer (cm)	pH	EC (μs·cm^-1^)	SOC (g·kg^-1^)	TN (g·kg^-1^)	TP (g·kg^-1^)	TK (g·kg^-1^)	AN (mg·kg^-1^)	AP (mg·kg^-1^)	AK (mg·kg^-1^)
**0–20**	8.42	156.35	12.59	0.80	0.80	23.86	97.66	16.48	81.53
**20–40**	8.54	138.23	9.53	0.75	0.62	23.74	75.63	3.42	71.63
**40–60**	8.78	124.25	8.67	0.60	0.59	21.92	49.47	2.65	52.84

**Table 2:** Basic information on different vegetation types in the alpine region of the Loess Plateau.

Vegetation type	Gradient (°)	Aspect	Altitude (m)	Previous land use type	De-farming time (years)	Mean DBH (cm)	Mean tree height (m)	Litter thickness (cm)
*Larix principis-rupprechtii*	10 ~ 25	Shady slope	2525 ~ 2530	Cropland	20	8.7	6.9	3.8
*Populus cathayana*	10 ~ 25	Shady slope	2465 ~ 2523	Cropland	20	11.9	7.2	4.5
*Picea crassifolia*	10 ~ 25	Shady slope	2509 ~ 2545	Cropland	20	5.1	3	0.7
*Betula platyphylla*	10 ~ 25	Shady slope	2517 ~ 2596	Cropland	20	4.0	2.1	0.6
Grassland	10 ~ 25	Shady slope	2511 ~ 2588	Cropland	20	—	—	0.9
Wheat field	5 ~ 10	Semi-shady slope	2478 ~ 2500	Cropland	—	—	—	—

Note: “―”means that this value does not exist.

### Field survey and soil sampling

Soil samples were collected from June to August 2018 for the experimental analysis. Each 30×30 m^2^ sample was evenly divided into nine 10×10 m^2^ small quadrats, and in each of the 10×10 m^2^ quadrat centers, a soil sampling point was established. At each soil sampling point, a soil auger with a diameter of 5 cm was used to take samples at 0–20 cm, 20–40 cm and 40–60 cm. Each sample was collected after removing litter, and the sampling point was at least 80 cm away from trees. Soil samples collected from each layer of the 9 sampling points were mixed into one sample. Approximately 100 g of mixed soil samples were taken back to the laboratory and placed in a well-ventilated area to dry naturally. All natural air-dried soil samples were filtered with a 2 mm mesh screen before the soil chemical property analysis.

In the four corners and center of each 30×30 m^2^ sample, a soil profile at 1 m long, 1 m wide and 60 cm deep was excavated. A core sampler with a diameter of 5 cm and a height of 5 cm was used to take three soil samples from the middle of each layer (0–20 cm, 20–40 cm, and 40–60 cm), and the samples were returned to the laboratory to measure the bulk density and porosity of the soil.

### Physical and chemical analysis

The bulk density and porosity of the soil were determined by the ring knife method. The soil pH and soil electrical conductivity (EC) were measured at a soil to water ratio of 1:5 (w/v) using pH and EC meters, respectively [11].

The SOC was determined by using the H_2_SO_4_-K_2_Cr_2_O_7_ oxidation method [10]. The soil total nitrogen (TN) and total phosphorus (TP) were determined by the Kjeldahl method after digestion with H_2_SO_4_ and molybdate ascorbic acid method after digestion with HClO_4_-H_2_SO_4_, respectively. The soil total potassium (TK) was determined using the flame photometer method after digestion with HF-HClO_4_ [44]. The soil hydrolyzed nitrogen (AN, the sum of ammonium nitrogen and nitrate nitrogen) was determined by the alkali diffusion method. The soil available phosphorus (AP) refers to phosphorus that can be absorbed by crops in the current season and includes all water-soluble phosphorus, as well as some adsorbed phosphorus and organic phosphorus [16]. The soil available potassium (AK) refers to potassium that can be absorbed and utilized by plants in the current season, including the water-soluble potassium and exchangeable potassium [11]. AP and AK were determined by the molybdenum antimony colorimetric method after extraction with NaHCO_3_ and the flame photometer method after extraction with CH_3_COONH_4_, respectively [25]. The soil SOC, TN, TP and TK contents are expressed as g·kg^-1^ on a dry weight basis, and the soil AN, AP and AK contents are expressed as mg·kg^-1^ on a dry weight basis. The stoichiometric ratios in soil were calculated by the mass ratio of SOC, TN, TP and TK.

### Statistical analysis

All variables are expressed as the mean and standard deviation. A multivariate analysis of variance was used to compare the SOC, TN, TP, TK, AN, AP, and AK contents and stoichiometric ratios of the soils under different vegetation restoration types and check the normality and uniformity of variance. A significance analysis was performed using the least significant difference (LSD) method. A Pearson correlation analysis was used to measure the correlation between variables. SPSS 24.0 and Origin 8.5 for Windows were used for the statistical analysis and to generate the figures, respectively.

## Results

### Soil physi-chemical properties

**Fig 2:**
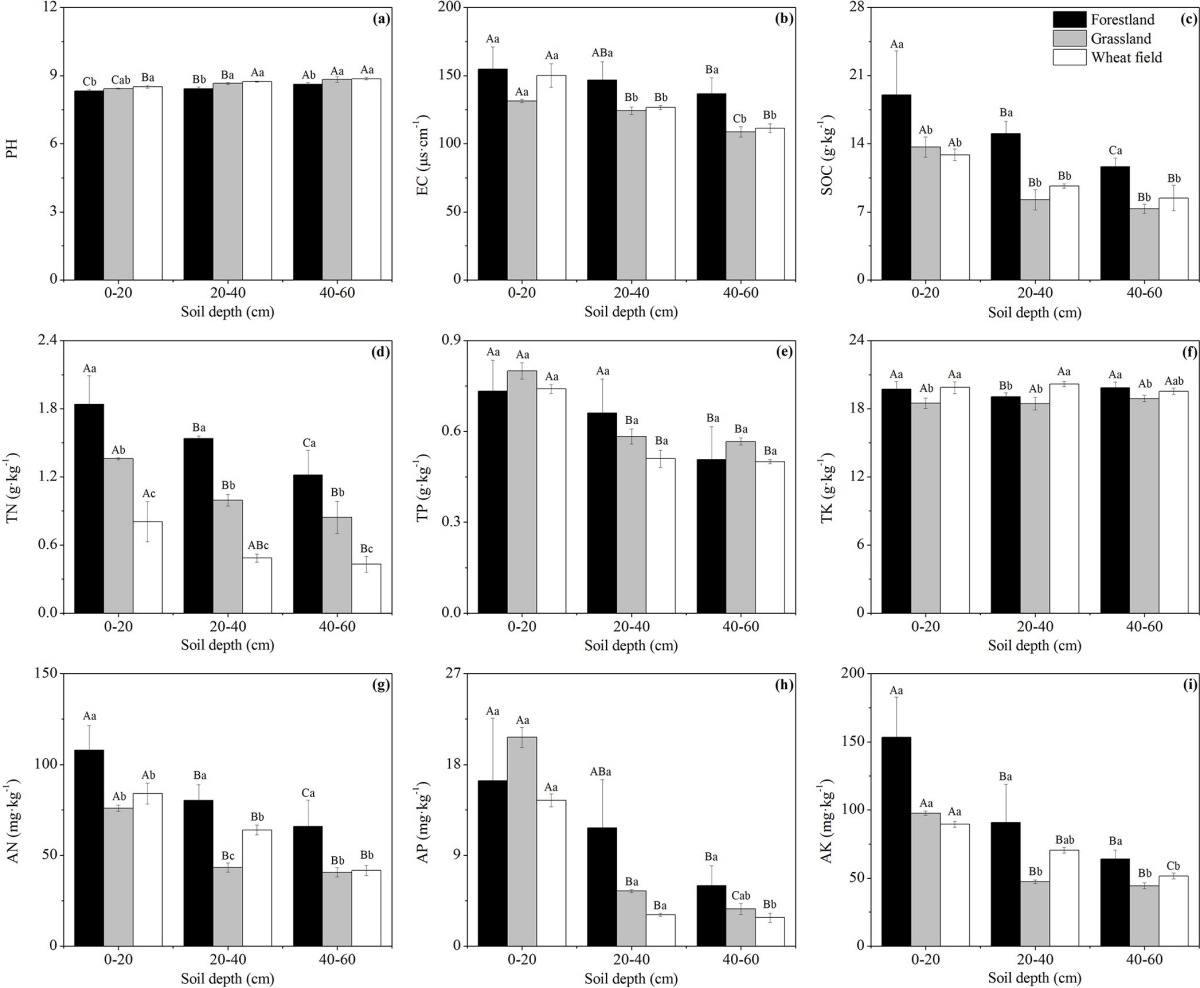
Profile distributions and contents of soil nutrients under different land use types. The error bars are the standard deviation of the mean. Different lowercase letters indicate significant differences at 0.05 (*P* < 0.05) levels among different land use types within the same soil layer. Different capital letters indicate significant differences at 0.05 (*P* < 0.05) levels in different soil layers of the same land use type.

In this study, the bulk density, total porosity and capillary porosity of different vegetation types were 1.1–1.4 g·cm^-3^, 40.9–56.5% and 38.1–52.2%, respectively (Table 3). WF had the highest bulk density and the lowest total porosity and capillary porosity in the 0–40 cm soil layer (*P* < 0.05). BP had the lowest bulk density and the highest total porosity and capillary porosity in the 0–20 cm soil layer (*P* < 0.05). LR had the lowest bulk density and the highest total porosity and capillary porosity in the 20–60 cm soil layer (*P* < 0.05). The pH and EC values among FL, GL and WF in this study ranged from 8.3 to 8.9 and from 108.8 μs·cm^-1^ to 154.8μs·cm^-1^, respectively (Fig 2A and 2B). The pH value of FL in all soil layers was lower than that of GL and WF, and the EC value was higher than that of GL and WF (*P* < 0.05). Moreover, the EC value of PR was higher than that of the other forest types in all soil layers and ranged from 152.2 to 174.8 μs·cm^-1^. In the different soil layers, the pH value was 0–20 cm < 20–60 cm, and the EC value was 0–20 cm > 20–60 cm.

**Table 3:** Bulk density and total porosity of different vegetation types.

Item	Soil layer (cm)	Forestland				Grassland	Wheat field	Significance coefficient of ANOVA
		*Picea crassifolia*	*Betula platyphylla*	*Larix principis-rupprechtii*	*Populus cathayana*			
**Bulk density (g.cm**^**−3**^**)**	0–20	1.30±0.07a	1.11±0.06b	1.27±0.08a	1.24±0.08a	1.30±0.05a	1.37±0.04a	0.014
	20–40	1.24±0.06bc	1.25±0.08bc	1.13±0.08c	1.27±0.06bc	1.39±0.13ab	1.44±0.06a	0.026
	40–60	1.32±0.05ab	1.28±0.05ab	1.20±0.09b	1.39±0.05a	1.40±0.10a	1.35±0.02a	0.042
**Total porosity (%)**	0–20	50.96±2.90ab	55.66±2.41a	53.55±3.32ab	50.66±3.02ab	50.11±1.88b	43.03±1.39c	0.007
	20–40	51.79±2.98ab	50.24±3.68ab	56.46±1.44a	50.45±2.75ab	46.91±4.33bc	40.92±2.51c	0.012
	40–60	47.51±3.46b	48.55±2.14ab	52.71±2.07a	43.93±2.24b	46.9±1.54b	45.81±0.21b	0.032
**Capillary porosity (%)**	0–20	46.66±1.31b	51.90±2.61a	49.26±0.74ab	46.48±4.14b	47.45±2.19ab	39.9±0.31c	0.008
	20–40	47.98±2.08ab	46.93±3.41ab	52.24±1.57a	43.41±5.93bc	43.86±4.17bc	38.13±1.50c	0.045
	40–60	44.17±2.96b	45.58±3.87ab	50.42±1.17a	40.29±1.32b	42.73±3.78b	43.14±0.57b	0.048

Note: The values in the table are the mean ± SD. Different lowercase letters in the table indicate significant differences among different vegetation types.

### Soil nutrient content and profile distribution

**Fig 3:**
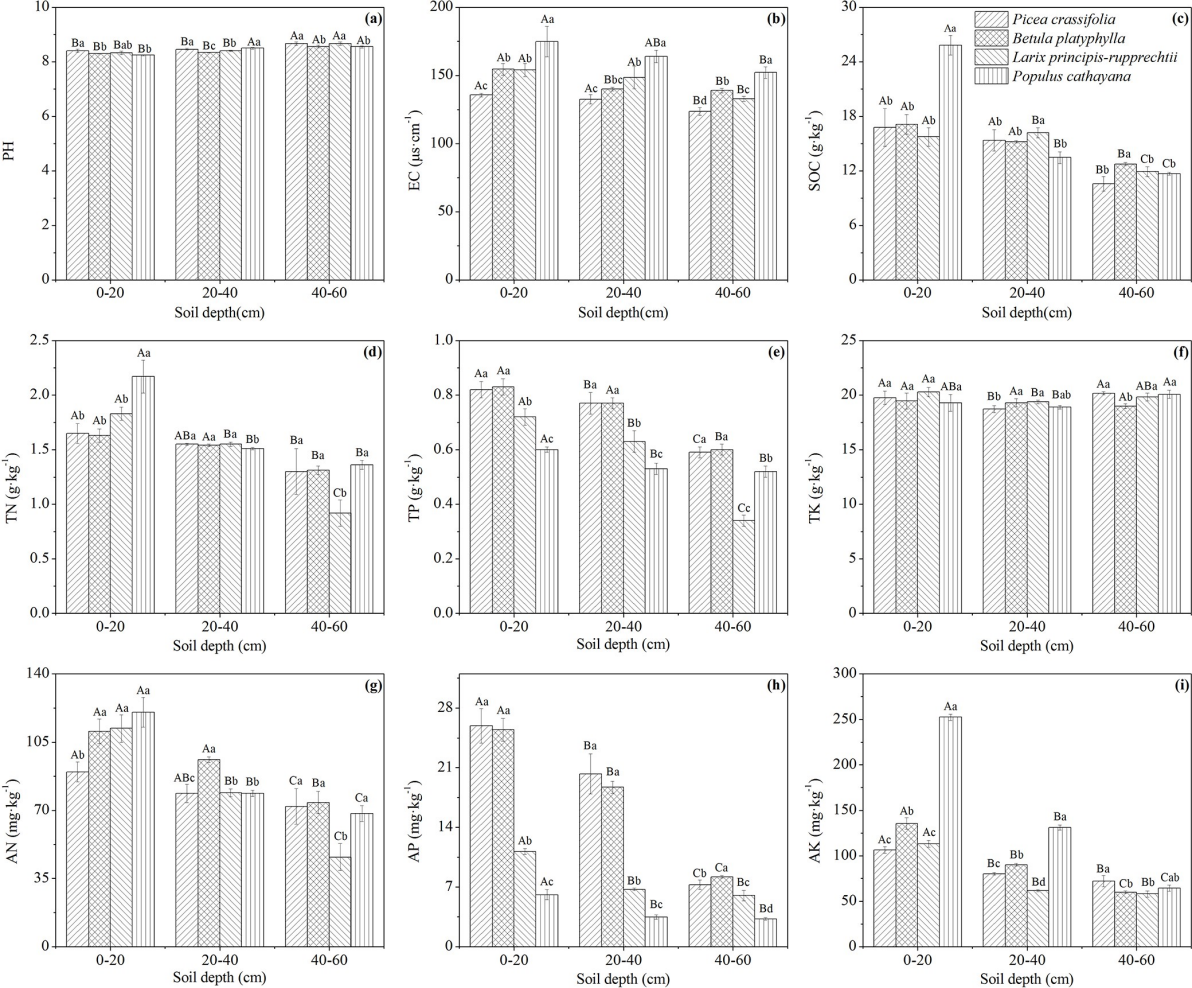
Profile distributions and contents of soil nutrients under different plantation types. The error bars are the standard deviation of the mean. Different lowercase letters indicate significant differences at 0.05 (*P* < 0.05) levels among different plantation types within the same soil layer. Different capital letters indicate significant differences at 0.05 (*P* < 0.05) levels in different soil layers of the same plantation type.

The soil nutrient content differed among the FL, GL and WF plots (Fig 2). At soil depths of 0–60 cm, the average SOC, TN, TP and TK contents of FL, GL and WF were as follows: 15.24 g·kg^-1^, 9.75 g·kg^-1^ and 10.32 g·kg^-1^, respectively (SOC); 1.53 g·kg^-1^, 1.07 g·kg^-1^ and 0.57 g·kg^-1^, respectively (TN); 0.63 g·kg^-1^, 0.65 g·kg^-1^ and 0.58 g·kg^-1^, respectively (TP); and 19.54 g·kg^-1^, 18.61 g·kg^-1^ and 19.86 g·kg^-1^, respectively (TK). The SOC, TN, AN and AK contents of FL in each soil layer were significantly higher than those of GL and WF (*P* < 0.05) (Fig 2C, 2D, 2G and 2I). GL had the lowest TK content in all soil layers, and the difference was significant between 0–20 cm and 40–60 cm (*P* < 0.05) (Fig 2F). The soil nutrient content of different plantation types also differed (Fig 3). The SOC, TN and AK contents in the 0–20 cm soil layer of PR were significantly higher than those of the other plantations (*P* < 0.05) (Fig 3C, 3D and 3I). The TP and AP contents of PC and BP in each soil layer were significantly higher than those of LR and PR (*P* < 0.05) (Fig 3E and 3H). The distributions of all soil nutrient contents (except STK) at different soil depths under all vegetation types were 0–20 cm > 20–60 cm.

### Soil nutrient stoichiometry and profile distribution

**Fig 4:**
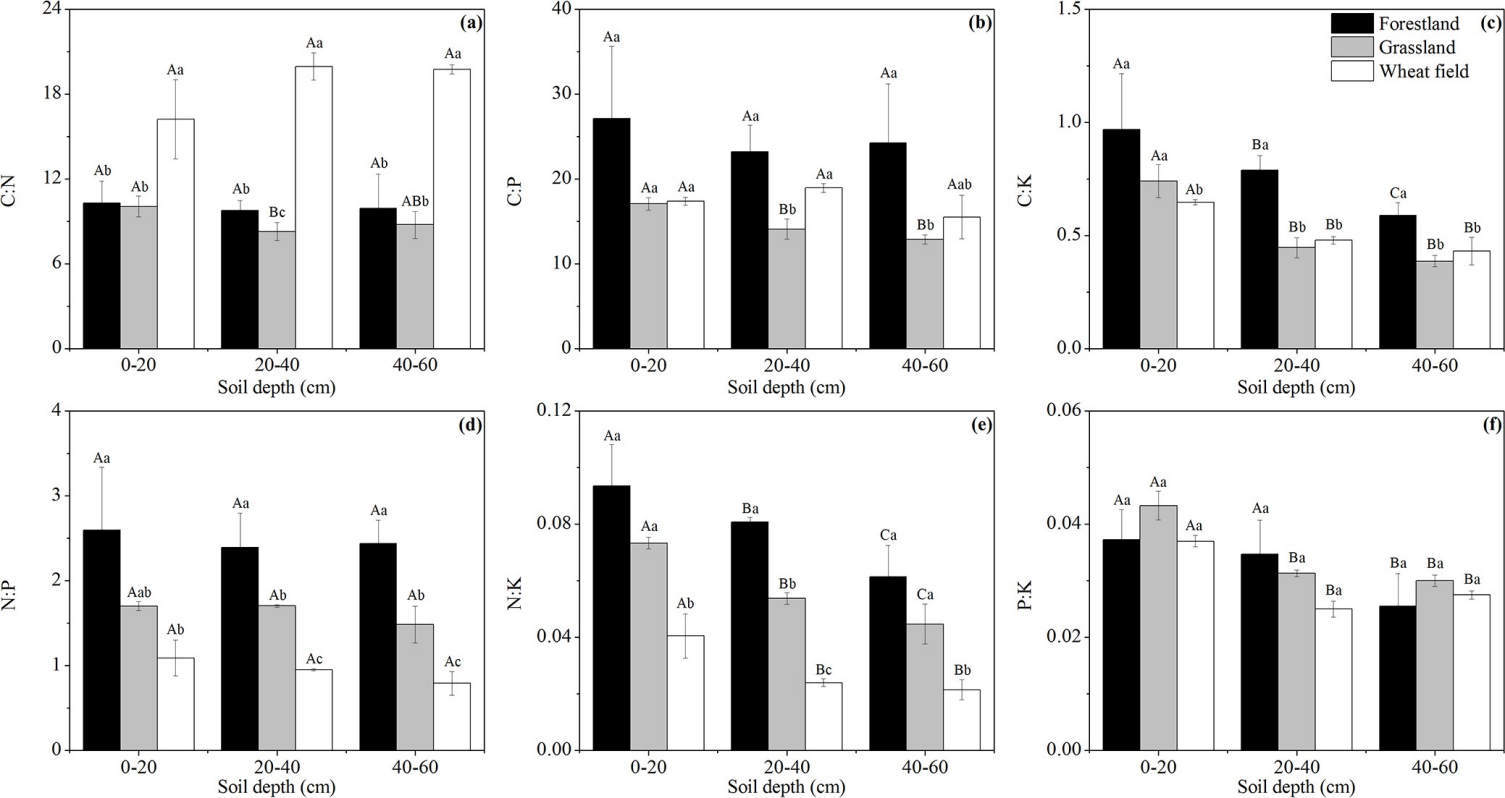
Profile distribution of soil nutrient stoichiometry under different land use types. The error bars are the standard deviation of the mean. Different lowercase letters indicate significant differences at 0.05 (*P* < 0.05) levels among different land use types within the same soil layer. Different capital letters indicate significant differences at 0.05 (*P* < 0.05) levels in different soil layers of the same land use type.

**Fig 5:**
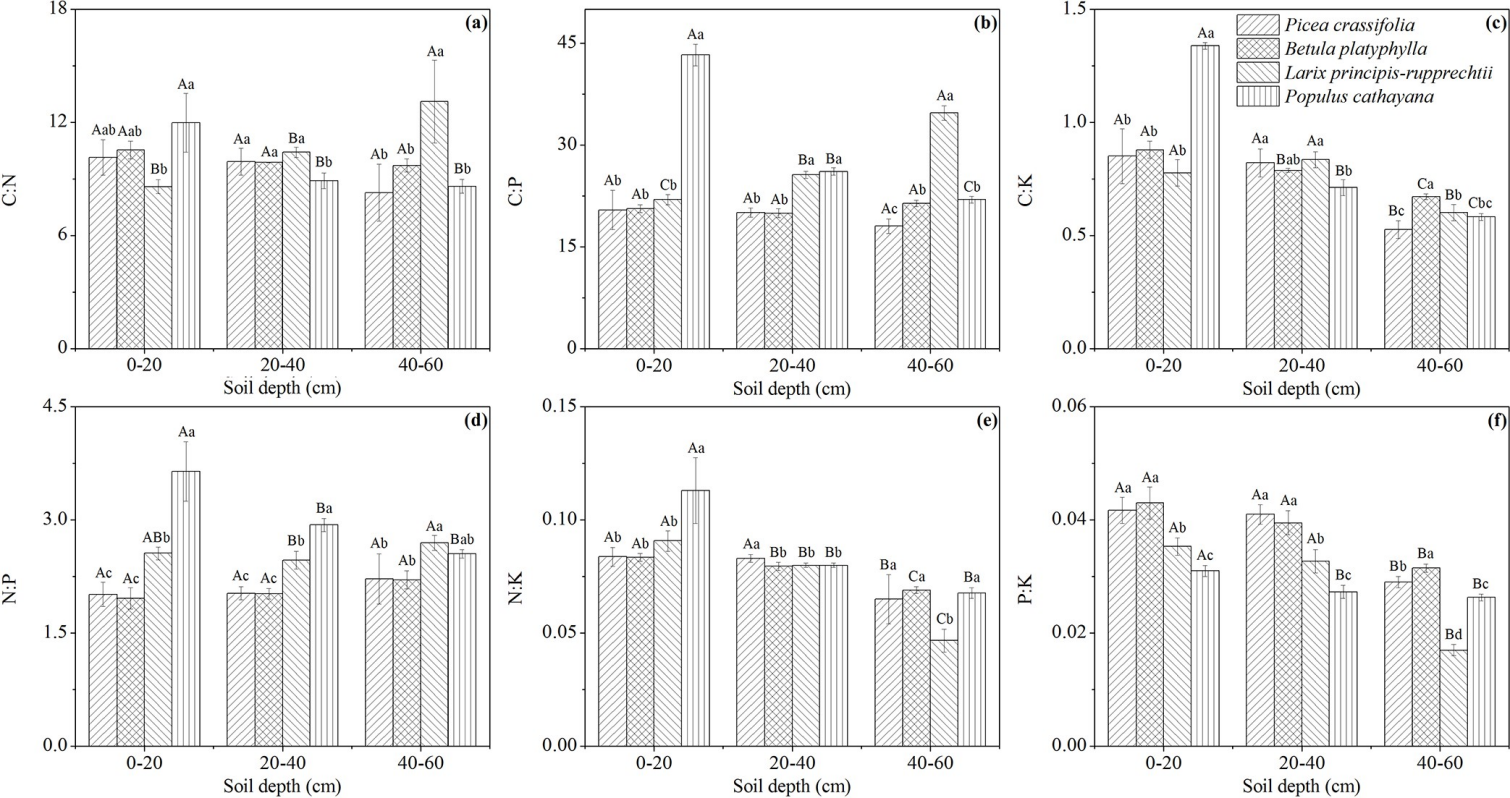
Profile distribution of soil nutrient stoichiometry under different plantation types. The error bars are the standard deviation of the mean. Different lowercase letters indicate significant differences at 0.05 (*P* < 0.05) levels among different plantation types within the same soil layer. Different capital letters indicate significant differences at 0.05 (*P* < 0.05) levels in different soil layers of the same plantation type.

The soil nutrient stoichiometry differed among the FL, GL and WF plots (Fig 4). The C:N ratio of WF in each soil layer was significantly higher than that of FL and GL (*P* < 0.05) (Fig 4A). The C:P and C:K ratios of FL in each soil layer were higher than those of GL and WF, and the differences were significant in the 40–60 cm soil layer (*P* < 0.05) (Fig 4B and 4C). The N:P and N:K ratios of each soil layer were ordered from FL > GL > WF, and the difference of the N:P ratio was significant (*P* < 0.05) (Fig 4D and 4E). The soil nutrient stoichiometry of different plantation types also differed (Fig 5). The soil stoichiometry (except P:K ratio) of PR at 0–20 cm was significantly higher than that of the other plantations (*P* < 0.05). LR had the highest C:N, C:P and N:P ratios and the lowest N:K and P:K ratios at 40–60 cm (*P* < 0.05). The P:K ratio of PC and BP in the 0–60 cm soil layer was significantly higher than that of LR and PR (*P* < 0.05) (Fig 5F). In the profile distribution of soil ecological stoichiometry, the soil stoichiometry of PR and GL (except the N:P ratio of GL) was 0–20 cm > 20–60 cm (*P* < 0.05), and the C:K, N:K and P:K ratios of WF were 0–20 cm > 20–60 cm (*P* < 0.05).

In this study, the soil C:N:P was between 12.9:1.5:1 and 43.3:3.6:1, and there were large differences among FL, GL and WF. The soil C:N:P of FL was higher than that of GL and WF. The soil C:N:P also differed according to plantation type. The soil C:N:P of PR and LR was higher than that of PC and BP (Table 4).

**Table 4:** C:N:P ratios of different vegetation types and soil depths.

Soil depth (cm)	*Picea crassifolia*	*Betula platyphylla*	*Larix principis-rupprechii*	*Populus cathayana*	Average value of forestland	Grassland	Wheat field	Average
**0–20**	20.4:2.0:1	20.6:2.0:1	22.0:2.6:1	43.3:3.6:1	26.6:2.6:1	17.1:1.7:1	15.7:1.1:1	19.8:1.8:1
**20–40**	20.1:2.0:1	19.9:2.1:1	25.7:2.5:1	26.1:2.9:1	23.0:2.4:1	14.1:1.7:1	19.0:1.0:1	18.7:1.7:1
**40–60**	18.1:2.2:1	21.5:2.2:1	34.7:2.7:1	21.9:2.5:1	24.1:2.4:1	12.9:1.5:1	15.5:0.8:1	17.5:1.6:1

### Relationships among the soil physi-chemical properties, nutrient content and stoichiometry

Between different soil physi-chemical properties and soil nutrients and stoichiometry, the bulk density and EC were strongly significantly correlated with the SOC, TN, C:P, C:K, N:P and N:K (*P* < 0.01) (Table 5). The total porosity, capillary porosity and pH were strongly significantly correlated with the SOC, TN and TP (*P* < 0.01) and significantly correlated with the soil stoichiometry (except for capillary porosity, which had no significant correlation with C:P) (*P* < 0.05). Moreover, pH had the closest correlation with the soil nutrients and stoichiometry among the soil physi-chemical properties. Between different soil nutrients and stoichiometry, the SOC, TN and TP had an extremely significant positive correlation (*P* < 0.01), and the correlation with SOC and TN was closer. SOC and TN were significantly correlated with soil stoichiometry (except for SOC, which had no significant correlation with C:N) (*P* < 0.05). SOC had the closest correlation with C:P and C:K, and TN had the closest correlation with C:N, N:P and N:K.

**Table 5:** Pearson correlation coefficient of soil properties, soil nutrient content and stoichiometry.

Item	SOC	TN	TP	TK	C:N	C:P	C:K	N:P	N:K	P:K
**Bulk density**	-0.670**	-0.704**	-0.262	0.012	0.252	-0.446**	-0.671**	-0.560**	-0.705**	-0.272
**Total porosity**	0.634**	0.746**	0.419**	-0.169	-0.413**	0.300*	0.648**	0.499**	0.758**	0.451**
**Capillary porosity**	0.546**	0.646**	0.378**	-0.196	-0.360*	0.244	0.567**	0.427****	0.666**	0.424**
**PH**	-0.859**	-0.872**	-0.578**	-0.026	0.300*	-0.464**	-0.853**	-0.559**	-0.862**	-0.556**
**EC**	0.762**	0.704**	0.112	0.161	-0.145	0.671**	0.741**	0.707**	0.685**	0.085
**SOC**	1	—	—	—	-0.062	0.737**	0.994**	0.647**	0.823**	0.357*
**TN**	0.828**	1	—	—	-0.568**	0.512**	0.827**	0.779**	0.994**	0.419**
**TP**	0.369**	0.428**	1	—	-0.196	-0.319*	0.381**	-0.214	0.422**	0.967**
**TK**	0.023	-0.016	-0.162	1	0.096	0.114	-0.059	0.061	-0.069	-0.251

Note

* means significant correlation (*P* < 0.05)

** means extremely significant correlation (*P* < 0.01); “―”denotes repetition.

The correlation analysis showed that pH, SOC and TN were most closely related to the soil nutrients and stoichiometry, indicating that the soil nutrients and stoichiometry were greatly affected by pH, SOC and TN.

## Discussion

### Effects of vegetation restoration patterns and soil depth on soil nutrient content

In our results, the SOC, TN, AN and AK contents in FL at all soil layers were significantly higher than those in GL and WF, and the SOC and TN contents in GL at the 0–20 cm soil layer were higher than those in WF, which is consistent with the results of Zhao [45] and Zhang [46]. The reasons for our results are as follows: (1) Soil nutrients are mainly derived from litter, senesced roots, root exudates and bioturbation [47]. After the implementation of the Grain-for-Green project, the litter of perennial vegetation returned to the soil, thereby increasing soil nutrients [48]. The contents of C and N are higher in litter [49], P is not easily decomposed [50], and water-soluble potassium is easily adsorbed and fixed on soil clay particles [48]; thus, a large increase in the SOC, TN, AN and AK contents was observed in the soil. Among the different land use types, FL had the highest litter biomass; therefore, it returned the most nutrients to the soil. (2) The root biomass in each soil layer exhibited the following order: forestland > grassland > cropland [22]. Compared with forestland and shrubbery, the roots of grassland and cropland are the shallowest and are mainly distributed in the topsoil layer [51]. Therefore, the residual root inputs in each soil layer of FL was the highest, and the residual root inputs in the 0–20 cm soil layer of GL was higher than that in WF. (3) At the same time, soil nutrients can be leached out by precipitation, and the aboveground parts and underground roots of plants can reduce soil erosion [2]. Therefore, the amount of soil nutrient loss in WF was higher than that in FL and GL. In our study, the AN content of WF was higher than that of GL in all soil layers, although the difference was not significant, which is inconsistent with the results of Zhang [46]. The main reason for this phenomenon is that local farmers use inorganic fertilizers in cropland to increase the daily yield of crops. After topsoil fertilization, AN will enter the deep soil after plowing and rainfall leaching. Therefore, the AN content of WF in the 0–60 cm soil layer in this study was higher than that of GL. In addition, each vegetation restoration pattern had lower bulk density and higher total porosity and capillary porosity in the 0–40 cm soil layer compared with WF. The contents of SOC, TN, AN and AK in each soil layer of FL and the contents of SOC and TN in the 0–20 cm soil layer of GL increased compared with those before restoration. The results showed that different vegetation restoration patterns yielded different degrees of improvement in soil properties and nutrients, and the conversion of farmland into forestland was more conducive to soil nutrient accumulation.

Among the different plantation types, the SOC, TN, AN and AK contents of PR in the 0–20 cm soil layer were higher than those of other plantations, which is consistent with the results of Shi [52]. Because PR belongs to broad-leaved forests, the soil C accumulation rate of the topsoil layer is higher [53, 54]. Moreover, the litter biomass of PR was the highest, and a humic layer was observed during the field investigation. Studies have shown that the process of humification in the soil is critical for ecosystems and plays an important role in improving soil fertility and storing C and N [55]. Therefore, compared with other plantations, PR had the highest input of organic matter in topsoil. Compared with the soil nutrients before restoration, the SOC, TN, AN and AK contents in the 0–20 cm soil layer of PR increased the most, which indicated that returning farmland to PR was beneficial to improving the surface soil nutrients.

The soil nutrient content (except TK) in this study was 0–20 cm > 20–60 cm, which is consistent with the results of previous studies [56]. The reason is that the surface soil is affected by the external environment, soil microorganisms, and nutrient return from the surface litter, which leads to a high content of nutrients in the surface soil [57]. After farmland was converted into forestland and grassland, the artificial flipping disappeared. With the increase in soil depth, the input of organic matter is limited by soil permeability, microbial decomposition activity and root absorption [58, 59], which reduces the nutrient content of deep soil.

### Effects of vegetation restoration patterns and soil depth on the soil nutrient stoichiometry

The study of ecological stoichiometry can help reveal the interaction between plants and soil [60]. Plants alter the soil ecological stoichiometry by influencing the soil nutrient content via nutrient uptake, litter inputs and root exudates [4]. Soil C:N reflects the decomposition rate of soil organic matter [61], and a lower C:N ratio corresponds to a faster decomposition rate [44]. In our study, the soil C:N ratio of each layer varied as follows: WF > FL > GL. Moreover, the difference between FL and GL was not significant in the 0–20 cm and 40–60 cm soil layers, which is consistent with the results of Zhou [62]. The reason is that soil organic matter is decomposed under the action of microorganisms, and litter can not only influence the structure of the soil microbial community and microbial biomass but also affect the decomposition process [8]. The higher the input of litter and residual roots, the more sufficient the substrate and the faster the decomposition rate [48]. The organic matter inputs in each soil layer of WF was the lowest; thus, the decomposition rate was the lowest and the C:N ratio was the highest. In addition, the C:N ratio of GL was higher than that of FL because litter in forestland has more biochemically recalcitrant materials (particularly aliphatic biopolymers), which are less suitable as microbial substrates compared with litter in grassland [63]; thus, the decomposition rate was slower than GL. In this study, the C:N ratio of PR in the 0–20 cm soil layer was significantly higher than that of the other plantations. The main reason is that organic matter releases C, N and P under the decomposition of microorganisms, but the microbial utilization efficiency of N and P is higher than that of C [16, 64]. Moreover, the uptake of N and P by plant roots leads to a greater accumulation of C in soil compared with N and P. However the biomass of PR litter was the highest, and the accumulation of C in surface soil was much greater than that of N; thus, the C:N ratio of PR was the highest.

In this study, the C:P, C:K, N:P and N:K ratios of FL in each soil layer were higher than GL and WF, and the N:P and N:K ratios of GL in each soil layer were higher than WF. The reason is that the TP and TK contents of FL, GL and WF in the 0–60 cm layer were not significantly different. Moreover, the correlation analysis showed that C:P and C:K had the closest correlation with SOC and N:P and N:K had the closest correlation with TN, indicating that C:P and C:K were mainly affected by SOC and N:P and N:K were mainly affected by TN, which is consistent with the results of Li [26]. In each soil layer, the SOC and TN contents of FL were significantly higher than those of GL and WF and the TN content of GL was higher than that of WF; thus, the highest C:P, C:K, N:P and N:K ratios were observed in FL and the N:P and N:K ratios of GL were higher than those of WF. Among the different plantation types, the C:P, C:K.N:P and N:K ratios of PR in the surface soil were significantly higher than those in the other plantations, which is consistent with the results obtained by Ren [65]. Because C:P and C:K were mainly affected by SOC, N:P and N:K were mainly affected by TN, and the SOC and TN of PR in topsoil were the highest; therefore, PR had the highest C:P, C:K, N:P and N:K ratios. The soil N:P ratio can determine the threshold of nutrient limitation. Vitousek [66] considered that N was the main factor affecting soil fertility when the ratio of soil C:N is less than 30 and the ratio of soil N:P is less than 14. The soil C:N ratio in this study ranged from 8.28 to 19.96, which is less than 30; the N:P ratio ranged from 0.79 to 2.60, which is less than 14. Therefore, N was the limiting element of soil fertility in the alpine region of the Loess Plateau. It is recommended to increase manure and crop residues in farmland to improve soil fertility and strengthen the application of N-fixing plants in different vegetation restoration patterns to increase the soil N content.

In different soil layers, the soil stoichiometry of PR and GL (except the N:P ratio of GL) was 0–20 cm > 20–60 cm, and the trend of other vegetation types was not significant. Because SOC and TN mainly affected the ecological stoichiometry, the soil SOC and TN contents were the highest at 0–20 cm; thus, the soil stoichiometry was higher in the surface layer. As the soil depth increases, root secretions and soil microorganisms are the main sources of soil nutrition. However, there are differences among different vegetation types; hence, the trend of other vegetation types is not significant.

## Conclusion

Different vegetation restoration patterns and soil depths had significant effects on the soil nutrients and stoichiometry in the alpine region of the Loess Plateau. FL increased the SOC, TN, AN and AK contents in each soil layer, while GL increased the TN content in each soil layer. Among the different plantation types, the SOC, TN, AN and AK contents in the topsoil layer of PR were the highest. In each soil layer, FL and GL reduced the C:N ratio, FL increased the C:P, C:K, N:P and N:K ratios, and GL increased the N:P and N:K ratios. Among the different plantation types, PR had the highest stoichiometry (except P:K ratio) in the topsoil layer. In addition, soil nutrients (except TK) at different depths were 0–20 cm > 20–60 cm. The soil stoichiometry of PR and GL (except the N:P ratio of GL) was 0–20 cm > 20–60 cm, and other vegetation types were not significant. Furthermore, the correlation analysis showed that SOC and TN had the strongest correlation with soil stoichiometry (except P:K ratio). These results indicated that different vegetation restoration patterns had different degrees of soil nutrient improvement. FL improved soil nutrients better than GL, and PR had the most significant improvement effect on surface soil nutrients. The soil fertility in this region was limited by N, and the soil stoichiometry was most affected by SOC and TN. To better understand the mechanism of vegetation restoration on soil nutrients and stoichiometry, soil enzyme activities and soil microorganisms can be studied in the future.

## Supporting information

S1 Table The detailed ANOVA results table for different land use types. Different lowercase letters indicate significant differences at 0.05 (*P* < 0.05) levels among different land use types within the same soil layer. Different capital letters indicate significant differences at 0.05 (*P* < 0.05) levels in different soil layers of the same land use type.
(PDF)

S2 Table The detailed ANOVA results table for different plantation types. Different lowercase letters indicate significant differences at 0.05 (*P* < 0.05) levels among different plantation types within the same soil layer. Different capital letters indicate significant differences at 0.05 (*P* < 0.05) levels in different soil layers of the same plantation type.
(PDF)
