# Supplementary material for: Soil C, N, P and K stoichiometry affected by vegetation restoration patterns in the alpine region of the Loess Plateau, Northwest China
Source: PLoS One. 2020 Nov 5;15(11):e0241859. doi: 10.1371/journal.pone.0241859 (PMC7644019; doi:10.1371/journal.pone.0241859)
Supplement: S1 Table — Different lowercase letters indicate significant differences at 0.05 (P < 0.05) levels among different land use types within the same soil layer. Different capital letters indicate significant differences at 0.05 (P < 0.05) levels in different soil layers of the same land use type. (PDF) [file pone.0241859.s001.pdf]

S1 Table. The detailed ANOVA results table for different land use types.

Item	Soil layer (cm)	Forestland			Grassland			Wheat field		
		Mean value	Standard deviation	Significant difference	Mean value	Standard deviation	Significant difference	Mean value	Standard deviation	Significant difference
pH	0-20	8.32	0.08	Cb	8.43	0.02	Cab	8.51	0.06	Ba
	20-40	8.43	0.06	Bb	8.65	0.04	Ba	8.73	0.03	Aa
	40-60	8.62	0.07	Ab	8.83	0.13	Aa	8.87	0.05	Aa
EC ($\mu s \cdot cm^{-1}$)	0-20	154.78	16.17	Aa	131.38	1.16	Aa	150.10	8.63	Aa
	20-40	146.78	13.34	ABa	124.28	2.76	Bb	126.65	1.48	Bb
	40-60	136.66	11.64	Ba	108.82	3.81	Cb	111.30	3.25	Bb
SOC ($g \cdot kg^{-1}$)	0-20	19.03	4.54	Aa	13.67	1.03	Ab	12.86	0.59	Ab
	20-40	15.05	1.27	Ba	8.25	1.05	Bb	9.66	0.24	Bb
	40-60	11.65	0.89	Ca	7.32	0.45	Bb	8.44	1.30	Bb
TN ($g \cdot kg^{-1}$)	0-20	1.84	0.25	Aa	1.36	0.01	Ab	0.81	0.18	Ac
	20-40	1.54	0.02	Ba	0.99	0.05	Bb	0.49	0.04	ABc
	40-60	1.22	0.22	Ca	0.84	0.14	Bb	0.43	0.07	Bc
TP ($g \cdot kg^{-1}$)	0-20	0.73	0.10	Aa	0.80	0.03	Aa	0.74	0.01	Aa
	20-40	0.66	0.11	Aa	0.58	0.03	Ba	0.51	0.03	Ba
	40-60	0.51	0.11	Ba	0.57	0.01	Ba	0.50	0.01	Ba

**TK (g·kg^{-1})**	0-20	19.72	0.67	Aa	18.49	0.45	Ab	19.86	0.53	Aa
	20-40	19.05	0.36	Bb	18.45	0.57	Ab	20.18	0.23	Aa
	40-60	19.84	0.51	Aa	18.90	0.27	Ab	19.54	0.29	Aab
**AN (mg·kg^{-1})**	0-20	107.91	13.54	Aa	76.00	1.73	Ab	84.00	5.66	Ab
	20-40	80.27	8.59	Ba	43.33	2.52	Bc	64.00	2.83	Bb
	40-60	65.91	14.51	Ca	40.67	2.52	Bb	41.67	2.83	Bb
**AP (mg·kg^{-1})**	0-20	16.40	6.20	Aa	20.70	0.98	Aa	14.45	0.64	Aa
	20-40	11.71	4.79	ABa	5.47	0.15	Ba	3.10	0.14	Ba
	40-60	6.00	1.96	Ba	3.70	0.56	Cab	2.83	0.46	Bb
**AK (mg·kg^{-1})**	0-20	153.27	29.55	Aa	97.67	1.53	Aa	89.50	2.12	Aa
	20-40	90.82	27.83	Ba	47.33	1.15	Bb	70.50	2.12	Bab
	40-60	63.91	6.77	Ba	44.33	2.08	Bb	51.50	2.12	Cb
**C:N**	0-20	10.30	1.57	Ab	10.07	0.73	Ab	16.24	2.79	Aa
	20-40	9.77	0.71	Ab	8.28	0.64	Bc	19.96	0.95	Aa
	40-60	9.95	2.41	Ab	8.77	0.95	ABb	19.76	0.33	Aa
**C:P**	0-20	27.12	10.50	Aa	17.07	0.71	Aa	17.37	0.47	Aa
	20-40	23.22	3.11	Aa	14.09	1.19	Bb	18.95	0.51	Aa
	40-60	24.27	6.94	Aa	12.89	0.56	Bb	15.53	2.57	Aab

**C:K**	0-20	0.97	0.25	Aa	0.74	0.07	Aa	0.65	0.01	Ab
	20-40	0.79	0.06	Ba	0.45	0.05	Bb	0.48	0.02	Bb
	40-60	0.59	0.06	Ca	0.39	0.03	Bb	0.43	0.06	Bb
**N:P**	0-20	2.60	0.74	Aa	1.70	0.05	Aab	1.09	0.21	Ab
	20-40	2.39	0.40	Aa	1.70	0.02	Ab	0.95	0.01	Ac
	40-60	2.44	0.27	Aa	1.48	0.22	Ab	0.79	0.14	Ac
**N:K**	0-20	0.09	0.01	Aa	0.07	0.00	Aa	0.04	0.01	Ab
	20-40	0.08	0.00	Ba	0.05	0.00	Bb	0.02	0.00	Bc
	40-60	0.06	0.01	Ca	0.04	0.01	Ca	0.02	0.00	Bb
**P:K**	0-20	0.04	0.01	Aa	0.04	0.00	Aa	0.04	0.00	Aa
	20-40	0.03	0.01	Aa	0.03	0.00	Ba	0.03	0.00	Ba
	40-60	0.03	0.01	Ba	0.03	0.00	Ba	0.03	0.00	Ba

Note: Different lowercase letters indicate significant differences at 0.05 ($P < 0.05$) levels among different land use types within the same soil layer. Different capital letters indicate significant differences at 0.05 ($P < 0.05$) levels in different soil layers of the same land use type.
