# Supplementary material for: Soil C, N, P and K stoichiometry affected by vegetation restoration patterns in the alpine region of the Loess Plateau, Northwest China
Source: PLoS One. 2020 Nov 5;15(11):e0241859. doi: 10.1371/journal.pone.0241859 (PMC7644019; doi:10.1371/journal.pone.0241859)
Supplement: S2 Table — Different lowercase letters indicate significant differences at 0.05 (P < 0.05) levels among different plantation types within the same soil layer. Different capital letters indicate significant differences at 0.05 (P < 0.05) levels in different soil layers of the same plantation type. (PDF) [file pone.0241859.s002.pdf]

S2 Table. The detailed ANOVA results table for different plantation types.

Item	Soil layer (cm)	*Picea crassifolia*			*Betula platyphylla*			*Larix principis-rupprechtii*			*Populus cathayana*		
		Mean value	Standard deviation	Significant difference	Mean value	Standard deviation	Significant difference	Mean value	Standard deviation	Significant difference	Mean value	Standard deviation	Significant difference
pH	0-20	8.41	0.06	Ba	8.30	0.01	Bb	8.33	0.06	Bab	8.24	0.02	Bb
	20-40	8.46	0.03	Ba	8.34	0.01	Bc	8.40	0.02	Bb	8.50	0.03	Aa
	40-60	8.67	0.06	Aa	8.56	0.04	Ab	8.67	0.06	Aa	8.55	0.04	Ab
EC (μs·cm^{-1})	0-20	135.7	1.26	Ac	154.5	4.38	Ab	154.03	4.89	Ab	174.78	11.15	Aa
	20-40	132.55	3.29	Ac	140.05	1.35	Bbc	148.38	8.2	Ab	163.88	4.51	ABa
	40-60	123.52	2.94	Bd	138.95	1.63	Bb	132.78	1.85	Bc	152.15	4.19	Ba
SOC (g·kg^{-1})	0-20	16.79	2.06	Ab	17.12	1.09	Ab	15.75	1.01	Ab	25.82	1.08	Aa
	20-40	15.37	1.17	Ab	15.22	0.12	Ab	16.19	0.57	Ba	13.46	0.65	Bb
	40-60	10.59	0.79	Bb	12.76	0.17	Ba	11.92	0.54	Cb	11.70	0.16	Cb
TN (g·kg^{-1})	0-20	1.65	0.09	Ab	1.63	0.06	Ab	1.83	0.06	Ab	2.17	0.15	Aa
	20-40	1.55	0.01	ABa	1.54	0.01	Aa	1.55	0.02	Ba	1.51	0.01	Bb
	40-60	1.30	0.21	Ba	1.31	0.04	Ba	0.92	0.12	Cb	1.36	0.04	Ba
TP (g·kg^{-1})	0-20	0.82	0.03	Aa	0.83	0.03	Aa	0.72	0.03	Ab	0.60	0.01	Ac
	20-40	0.77	0.04	Ba	0.77	0.02	Aa	0.63	0.04	Bb	0.53	0.02	Bc
	40-60	0.59	0.02	Ca	0.60	0.02	Ba	0.34	0.02	Cc	0.52	0.02	Bb

**TK (g·kg^{-1})**	0-20	19.76	0.61	Aa	19.47	0.74	Aa	20.28	0.43	Aa	19.30	0.78	ABa
	20-40	18.72	0.31	Bb	19.31	0.37	Aa	19.38	0.17	Ba	18.89	0.17	Bab
	40-60	20.16	0.14	Aa	18.99	0.23	Ab	19.84	0.36	ABa	20.08	0.37	Aa
**AN (mg·kg^{-1})**	0-20	89.67	5.13	Ab	110.50	6.36	Aa	112.00	7.00	Aa	120.33	7.64	Aa
	20-40	78.67	4.62	ABc	96.00	1.41	Aa	79.00	2.00	Bb	78.67	1.53	Bb
	40-60	72.00	9.17	Ca	74.00	5.66	Ba	46.00	6.93	Cb	68.33	4.04	Ca
**AP (mg·kg^{-1})**	0-20	25.90	2.01	Aa	25.45	1.34	Aa	11.17	0.35	Ab	6.10	0.60	Ac
	20-40	20.27	2.34	Ba	18.70	0.71	Ba	6.73	0.15	Bb	3.47	0.25	Bc
	40-60	7.27	0.55	Cb	8.20	0.14	Ca	6.00	0.62	Bc	3.27	0.15	Bd
**AK (mg·kg^{-1})**	0-20	106.33	3.79	Ac	135.50	6.36	Ab	113.00	3.61	Ac	252.33	3.51	Aa
	20-40	80.33	1.53	Bc	90.00	1.41	Bb	61.67	1.15	Bd	131.00	2.65	Ba
	40-60	72.00	6.25	Ba	60.00	1.50	Cb	58.00	3.46	Bb	64.33	3.51	Cab
**C:N**	0-20	10.14	0.94	Aab	10.54	0.47	Aab	8.60	0.36	Bb	11.99	1.57	Aa
	20-40	9.92	0.71	Aa	9.88	0.01	Aa	10.42	0.27	Ba	8.91	0.41	Bb
	40-60	8.27	1.51	Ab	9.73	0.35	Ab	13.10	2.19	Aa	8.61	0.37	Bb
**C:P**	0-20	20.44	2.91	Ab	20.63	0.59	Ab	21.96	0.74	Cb	43.27	1.58	Aa
	20-40	20.05	0.71	Ab	19.97	0.67	Ab	25.67	0.52	Ba	26.10	0.53	Ba
	40-60	18.05	1.08	Ac	21.42	0.45	Ab	34.72	1.09	Aa	21.95	0.51	Cb

**C:K**	0-20	0.85	0.12	Ab	0.88	0.04	Ab	0.78	0.06	Ab	1.34	0.01	Aa
	20-40	0.82	0.06	Aa	0.79	0.01	Bab	0.84	0.04	Aa	0.71	0.04	Bb
	40-60	0.53	0.04	Bc	0.67	0.01	Ca	0.60	0.04	Bb	0.58	0.01	Cbc
**N:P**	0-20	2.01	0.16	Ac	1.96	0.14	Ac	2.56	0.09	ABb	3.64	0.40	Aa
	20-40	2.03	0.09	Ac	2.02	0.07	Ac	2.47	0.11	Bb	2.93	0.09	Ba
	40-60	2.22	0.33	Ab	2.21	0.12	Ab	2.70	0.10	Aa	2.55	0.06	Bab
**N:K**	0-20	0.08	0.00	Ab	0.08	0.00	Ab	0.09	0.00	Ab	0.11	0.01	Aa
	20-40	0.08	0.00	Aa	0.08	0.00	Bb	0.08	0.00	Bb	0.08	0.00	Bb
	40-60	0.07	0.01	Ba	0.07	0.00	Ca	0.05	0.01	Cb	0.07	0.00	Ba
**P:K**	0-20	0.04	0.00	Aa	0.04	0.00	Aa	0.04	0.00	Ab	0.03	0.00	Ac
	20-40	0.04	0.00	Aa	0.04	0.00	Aa	0.03	0.00	Ab	0.03	0.00	Bc
	40-60	0.03	0.00	Bb	0.03	0.00	Ba	0.02	0.00	Bd	0.03	0.00	Bc

Note: Different lowercase letters indicate significant differences at 0.05 ($P < 0.05$) levels among different plantation types within the same soil layer. Different capital letters indicate significant differences at 0.05 ($P < 0.05$) levels in different soil layers of the same plantation type.
